# Mitigation of Gastric Damage Using *Cinnamomum cassia* Extract: Network Pharmacological Analysis of Active Compounds and Protection Effects in Rats

**DOI:** 10.3390/plants11060716

**Published:** 2022-03-08

**Authors:** Ji Hwan Lee, Hee Jae Kwak, Dongchul Shin, Hye Jin Seo, Shin Jung Park, Bo-Hee Hong, Myoung-Sook Shin, Seung Hyun Kim, Ki Sung Kang

**Affiliations:** 1 Cooperative-Center of Natural Product Central Bank for Biological Evaluation, College of Korean Medicine, Gachon University, Seongnam 13120, Korea; kleert26@gmail.com (J.H.L.); sdc2510@gmail.com (D.S.); ms.shin@gachon.ac.kr (M.-S.S.); 2 Yonsei Institute of Pharmaceutical Sciences, College of Pharmacy, Yonsei University, Incheon 21983, Korea; moon3685@naver.com; 3 Chong Kun Dang (CKD) Pharm Research Institute, Yongin-si 16995, Korea; hyejins@ckdpharm.com (H.J.S.); parksj@ckdpharm.com (S.J.P.); hongbh@ckdpharm.com (B.-H.H.)

**Keywords:** *Cinnamomum cassia*, acute gastric injury, EtOH/HCl mixture, indomethacin, network pharmacological analysis

## Abstract

Gastritis is a common disease worldwide that is caused by various causes such as eating habits, smoking, severe stress, and heavy drinking, as well as *Helicobacter pylori* infections and non-steroidal anti-inflammatory drugs. *Cinnamomum cassia* is a tropical aromatic evergreen tree commonly used as a natural medicine in Asia and as a functional food ingredient. Studies have reported this species’ anti-obesity, anti-diabetic, and cardiovascular disease suppression effects. We evaluated the potential effects of *C. cassia* using non-steroidal anti-inflammatory drugs (NSAIDs), ethanol (EtOH), and ethanol/hydrochloric acid (HCl)-induced gastric mucosal injury models. *C. cassia* extracts reduced the area of gastric mucosa injury caused by indomethacin, NSAID, EtOH, and EtOH/HCl. We also applied a network pharmacology-based approach to identify the active compounds, potential targets, and pharmacological mechanisms of *C. cassia* against gastritis. Through a network pharmacology analysis, 10 key components were predicted as anti-gastritis effect-related compounds of *C. cassia* among 51 expected active compounds. The NF-κB signaling pathway, a widely known inflammatory response mechanism, comprised a major signaling pathway within the network pharmacology analysis. These results suggest that the anti-gastritis activities of *C. cassia* may be induced via the anti-inflammatory effects of key components, which suppress the inflammation-related genes and signaling pathways identified in this study.

## 1. Introduction

Gastritis is a common disease that affects many people every year worldwide. A gastrointestinal mucosal injury is caused by environmental factors such as eating habits, smoking, severe stress, and heavy drinking, as well as *Helicobacter pylori* infections and non-steroidal anti-inflammatory drugs (NSAIDs, including indomethacin and aspirin) [1,2]. This inflammatory disease of the gastrointestinal mucosa is called gastritis, and damage to the mucous membrane progresses and can develop into chronic gastritis and gastric ulcers, which ultimately become the cause of gastric cancer [3]. Common symptoms of gastritis include a burning pain in the upper abdomen, indigestion, vomiting, and nausea [4]. In addition, gastritis caused by a *Helicobacter pylori* infection increases gastric acid secretion [5]. Gastric acid secretion is induced by histamine 2 receptors (H2r) that bind to histamine and secrete gastric acid into the stomach lumen, which is then accelerated by the cAMP/protein kinase A/proton pump pathway [6]. Thus, most gastritis and gastric ulcer treatments use histamine 2-receptor agonist (H2RA)-type drugs such as ranitidine and cimetidine, as well as proton pump inhibitor (PPI)-type drugs such as pantoprazole and lansoprazole [7,8]. Although these drugs have demonstrated an acceptable clinical efficacy, side effects such as rashes and urticarial and digestive disorders have been reported [9,10]. Therefore, the development of therapeutic agents for gastritis and gastric ulcers using natural products, with no side effects, is in progress. Several studies have recently reported the protective effects of natural products without side effects and toxicity against gastric mucosal damage using several animal models [9,11,12].

*Cinnamomum cassia* (*C. cassia*) is a tropical aromatic evergreen tree commonly used in Asian medicine (including Korea, China, and India) and food ingredients [13]. In previous studies, *C. cassia* has demonstrated antitumor, anti-inflammatory, analgesic, antidiabetic, antiobesity, antibacterial, antiviral, and cardiovascular pharmacological effects [13,14]. In addition, the components of this herb have been identified, including 64 terpenoids, 16 phenylpropanoids, 24 glycosides, 26 lignans, and 9 lactones [13]. An efficacy evaluation of *C. cassia* in the stomach in treating mild convulsive gastrointestinal disorders was reported by the European Medicines Agency in 2011, and the beneficial effects of oral administration on gastric mucosal damage were investigated [15]. This herb and its compounds have reportedly exerted anti-inflammatory effects by inhibiting IL-8 in *Helicobacter pylori* [16]. In this study, we investigated the potential effects of *Cinnamomum cassia* extracts using an in vivo model of acute gastric mucosal injury induced by indomethacin, EtOH, and EtOH/HCl.

Network pharmacology has evolved through the convergence of bioinformatics, systems biology, and network analysis. Recently, this field has attracted attention as a new potential approach for interpreting the traditional efficacy of natural products. For example, Xiaona et al. identified the active ingredients of *Sparganii rhizome* and its anti-cancer mechanism in treating gastric cancer using network pharmacology analysis [17]. Additionally, Asi et al. identified the mechanisms of *Artemisiae scoparia* in treating chronic hepatitis B using a network pharmacology approach [18]. Network pharmacology thus provides a new opportunity to infer the active compounds and pharmacological mechanisms of natural products effectively.

An experimental validation was performed in the present study to confirm the anti-gastritis effects of the *C. cassia* extract in rats. Moreover, we applied a network pharmacology-based approach to deduce the active compounds, potential targets, and pharmacological mechanisms of standardized extracts of *C. cassia* in treating gastritis.

## 2. Results

### 2.1. Effect of C. cassia Extracts on Indomethacin Induced Gastric Ulcers

**Figure 1:**
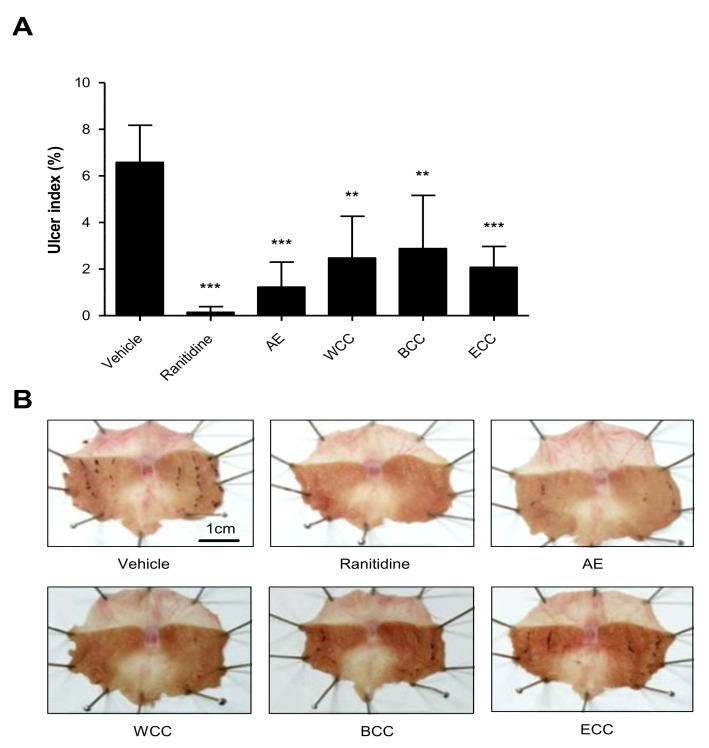
Comparison in the efficacy of *C. cassia* extract in treating gastric lesions. The test articles of *C. cassia* were pretreatment by oral administration. After 30 min, indomethacin was administered orally. After 5 h, a rat was sacrificed, and its ulcers were measured. (**A**) Configuration demonstrating the effects of the following vehicles. Positive controls: ranitidine (75 mg/kg, positive control) and AE (150 mg/kg, *Artemisia* extract, positive control), and with various *C. cassia* extracts: WCC (150 mg/kg, water extract of *C. cassia*), BCC (150 mg/kg, water extract *C. cassia* pre-washed with butanol), ECC (150 mg/kg, water extract of *C. cassia* pre-washed with ethyl acetate). (**B**) Representative effects on indomethacin-induced gastric lesions in various treatment groups. The results are expressed in mean ± SD (*n* = 10); statistical analysis was performed using SPSS 19, and *p*-values were set at level *p* < 0.01 (**) and *p* < 0.001 (***).

Gastric mucosal injuries caused by indomethacin were confirmed in the vehicle group. The injured area comprised 6.58% ± 1.60 of the total gastric mucosal area. The gastric mucosa injury area of each group with treated *C. cassia* extracts numbered 2.48% ± 1.79 of the total gastric mucosa area in WCC (water extract of *C. cassia*), 2.88% ± 2.28 of the total gastric mucosa area in BCC (water extract of *C. cassia* pre-washed with butanol), and 2.07% ± 0.91 of the total gastric mucosa area in ECC (water extract of *C. cassia* pre-washed with ethyl acetate). In the positive control group, the gastric mucosa injury area was observed 0.20% ± 0.29 of the total gastric mucosa area in ranitidine, and 1.23% ± 1.07 of the total gastric mucosa area in an *Artemisia* extract (AE) (Figure 1). ECC was lowered considerably more than in the other extracts; however, this difference was not statistically significant.

**Figure 2:**
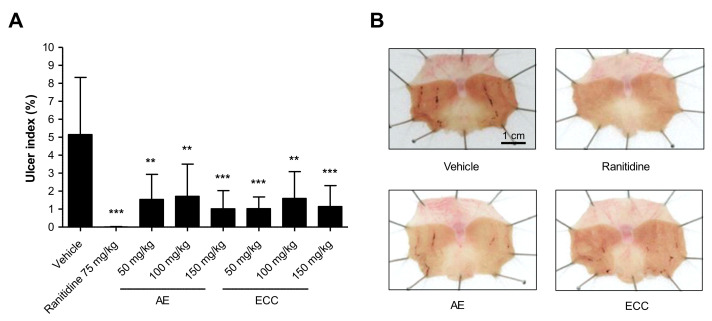
Protective effects of *C. cassia* extract against gastric damage. (**A**) Inhibiting effects of AE and ECC on ulcer indexes in SD rats. The ulcer index percentages showed markedly reduced gastric damage in each treatment concentration. (**B**) Representative comparison of gastric injury in the four studied groups. Data compared % of vehicle. ** *p* < 0.01, *** *p* < 0.001.

In the experiment, in order to confirm the dose-dependent effects of AE and ECC, the gastric mucosa injury area of each group treated with AE totaled 1.54% ± 1.39 of in the group treated with 50 mg/kg, 1.71% ± 1.79 in the group treated with 100 mg/kg, and 1.03% ± 1.31 in the group treated with 150 mg/kg. The gastric mucosa injury areas of each group treated with ECC were 1.03% ± 0.65 in the group treated with 50 mg/kg, 1.59% ± 1.49 in the group treated with 100 mg/kg, and 1.14% ± 1.33 in the group treated with 150 mg/kg (Figure 2). Therefore, the protective effect of ECC was as strong as that of AE.

### 2.2. Effect of C. cassia Extracts on Gastric Mucosa Injury Using EtOH

We compared the effects of *C. cassia* extracts on HCl-EtOH- and EtOH-induced gastric mucosal injuries. The HCl/EtOH mixture damaged 8.44% ± 3.84 of the total gastric mucosal area in the vehicle group. The ranitidine-treated group had an inhibitory effect of 15.78% compared with the vehicle group. The 75 mg/kg ranitidine-treated group demonstrated an inhibitory effect of 15.78% compared to the vehicle group. Each group treated with *C. cassia* extracts showed higher inhibitory effects than the ranitidine-treated group; an 18.09% inhibition was observed in the 150 mg/kg WCC-treated group, with a 44.46% inhibition in the 150 mg/kg ECC-treated group. However, these inhibitory effects were not statistically significant.

The 100% EtOH mixture showed a higher gastric mucosa injury than using the HCl/EtOH mixture damage. In addition, ranitidine did not inhibit gastric mucosal injuries. However, the *C. cassia* extract article showed a 27.74% inhibition in the 150 mg/kg WCC-treated group and a 29.74% inhibition in the 150 mg/kg ECC-treated group (Table 1). However, these inhibitory effects were not statistically significant.

### 2.3. Effect of C. cassia Extracts on HCl-EtOH-Induced Changes in Volume, pH, and Total Acidity

Next, the efficacy of the *C. cassia* extract in treating gastric juice secretions was confirmed. The HCl/EtOH mixture was used to induce gastritis, and gastric juice secretions were evaluated by measuring the gastric juice volume, pH, and gastric acidity. The gastric juice volume in the control group was higher than the vehicle group, with 0.6 mL ± 0.5 in the latter and 3.5 mL ± 0.7 in the former. Additionally, the gastric juice volumes of the treatment groups decreased compared to the control group, with 3.4 mL ± 0.7 in the group treated with 30 mg/kg Lansoprazole, 2.8 mL ± 0.7 in the group treated with 300 mg/kg WCC, and 2.7 mL ± 0.6 in the group treated with 300 mg/kg ECC. ECC significantly inhibited the increase in gastric juice volume induced by HCl-EtOH.

The gastric juice pH in the control group was higher than in the vehicle group, with 2.1 ± 0.8 in the latter and 3.7 ± 1.0 in the former. The gastric juice pH of each treatment group decreased compared to the control group, with 2.9 ± 0.9 in the group treated with 300 mg/kg WCC and 3.6 ± 0.5 in the group treated with 300 mg/kg ECC. However, lansoprazole increased the pH to 6.2 ± 1.0.

The total gastric acidity in the control group was higher than that in the vehicle group (0.040 mEq/4 h ± 0.041 in the latter, and 0.112 mEq/4 h ± 0.055 in the former). The total gastric acidity of each treatment group was lower than that of the control group (0.030 mEq/4 h ± 0.018 in the group treated with 30 mg/kg lansoprazole, 0.101 mEq/4 h ± 0.024 in the group treated with 300 mg/kg WCC, and 0.087 mEq/4 h ± 0.017 in the 300 mg/kg ECC-treated group) (Table 2).

### 2.4. Network Pharmacology Analysis

#### 2.4.1. Drug-Likeness (DL) and Oral Bioavailability (OB) Screening of Chemical Components

A total of 114 chemical components of *C. cassia* were identified from reviewed databases and the literature. All collected compounds were evaluated for DL and OB using the QED method to select the expected active compounds (Table S1). Eight physicochemical properties were obtained from the SwissADME database to calculate QED value: (1) molecular weight (MW), (2) octanol–water partition coefficient (ALOGP), (3) number of hydrogen bond acceptors (HBA), (4) number of hydrogen bond donors (HBD), (5) polar surface area (PSA), (6) number of rotatable bonds (ROTB), (7) number of aromatic rings (AROM), and (8) number of structural alerts (ALERT). OB was judged on the basis of Veber’s rule: (1) ROTB of 10 or less, (2) sum of HBA and HBD of ≤12, and (3) PSA of 140 or less. If the above rule was satisfied, the OB value was marked as TRUE; otherwise, it was marked as FALSE. The cut-off values of QED and OB for the selection of expected active compounds were set to 0.4 or more and TRUE, respectively. On the basis of these cut-off values, 94 compounds were screened as expected active compounds (Table S2).

#### 2.4.2. Prediction of Targets and Identification of Potential Targets

**Figure 3:**
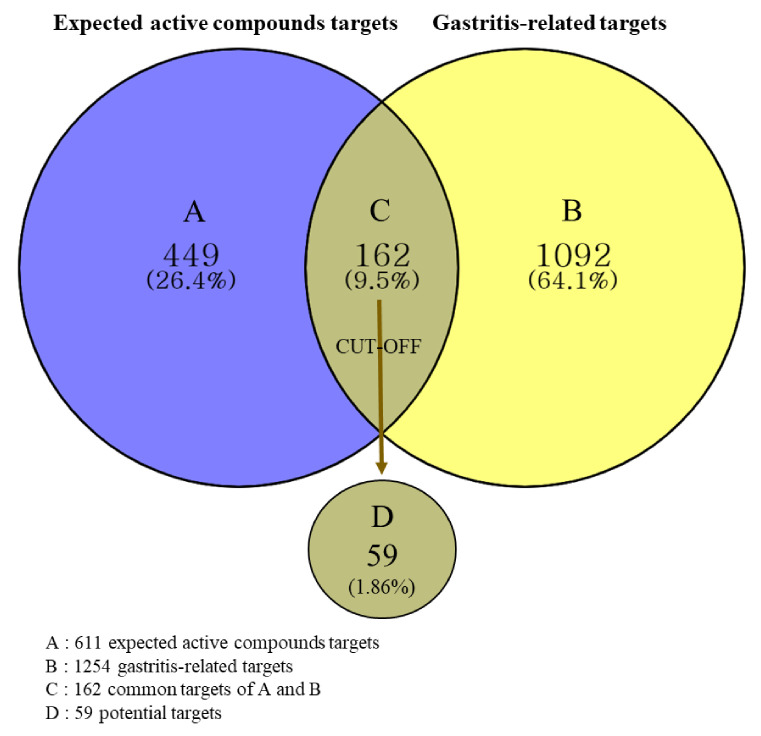
Venn diagram of expected active compounds and gastritis-related targets.

Targets for the expected active compounds were obtained using the SwissTargetPrediction database. A total of 611 expected active compound targets were identified after removing duplicates and false-positive targets. In total, 1254 gastritis-related targets were acquired from the GeneCards database. As shown in Figure 3, 162 targets were intersected, and a relevance score (≥1000) was used as the cut-off value to select potential targets. Ultimately, 59 potential targets were selected (Table 3).

#### 2.4.3. Construction and Analysis of Protein–Protein Interaction (PPI) Networks of Potential and Key Targets

**Figure 4:**
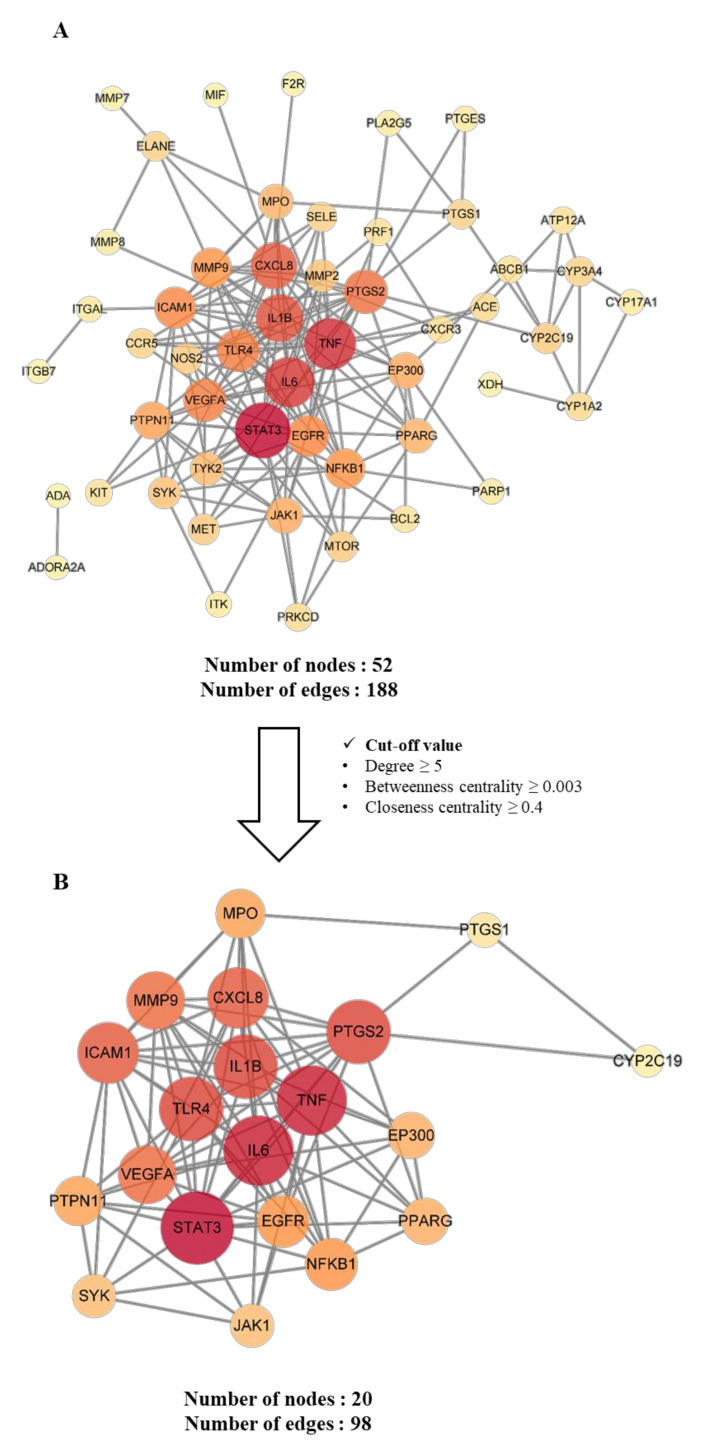
Protein–protein interaction (PPI) networks: (**A**) PPI network of potential targets; (**B**) PPI network of the key targets.

PPI networks were established to ensure a comprehensive understanding of the interactions between the target genes. The STRING database was used to gather interactions between potential target genes. The interaction data were submitted to the Cytoscape software to construct PPI networks. Topological network analysis was performed using three major topological analytic parameters: degree (degree centrality), betweenness centrality, and closeness centrality. These parameters indicate the importance of nodes within a network. Among these three parameters, degree is the most intuitive evaluation index, which is defined as the number of node connections. Thus, high degree values represent the significance of the nodes in a network [19]. Betweenness centrality is defined as the number of shortest paths between nodes. Specifically, it helps identify the nodes that bridge others within the network [20]. Closeness centrality specifies the length of the shortest path between two nodes. In other words, a high closeness centrality means that it has close relationships with many nodes [21]. As shown in Figure 4, PPI networks were constructed; the nodes represent the target genes, and the edges indicate the links between target genes. The size and color of a node indicate the intensity of its degree. Thus, the higher the degree of the node, the larger the node, and the color gradually deepens from yellow to red. The width of the edge indicates the degree of correlation between targets. In other words, the larger the combined score, the higher the degree of binding between nodes, and the thicker the edge.

As shown in Figure 4A, the PPI network of the potential targets consisted of 52 nodes and 188 edges. Topological network analysis was performed to identify key targets. The three parameters were set as cut-off values: degree ≥5, betweenness centrality ≥0.003, and closeness centrality ≥0.4; 20 targets remained following the input of the cut-off values. As shown in Figure 4B, the PPI network of key targets consisted of 20 nodes and 98 edges. Among the 20 key targets, STAT3 (signal transducer and activator of transcription 3), IL6 (interleukin 6), TNF (tumor necrosis factor), IL1B (interleukin 1 beta), TLR4 (Toll-like receptor 4), PTGS2 (prostaglandin-endoperoxide synthase 2), CXCL8 (C-X-C motif chemokine ligand 8), IAM1 (intercellular adhesion molecule 1), MMP9 (matrix metallopeptidase 9), and VEGFA (vascular endothelial growth factor A) showed >10 degrees (Table 4). Specifically, IL6, TNF, IL1B, and CXCL8 exhibited relevance scores of ≥10. These targets comprise both widely known inflammatory mediators and successful targets for controlling inflammation [22,23,24,25]. Furthermore, these genes are also important in controlling gastritis. *Helicobacter pylori* (*H. pylori*), one of the causes of gastritis, induces inflammatory responses mediated by pro-inflammatory cytokines, such as IL6 [26]. Moreover, the expression levels of TNF, IL1B, and CXCL8 increase in the gastric mucosa during a *H. pylori* infection [27,28]. Therefore, these results suggest that key targets might be crucial in treating gastritis.

#### 2.4.4. Gene Ontology (GO) and Kyoto Encyclopedia Genes and Genomes (KEGG) Pathway Enrichment Analysis

**Figure 5:**
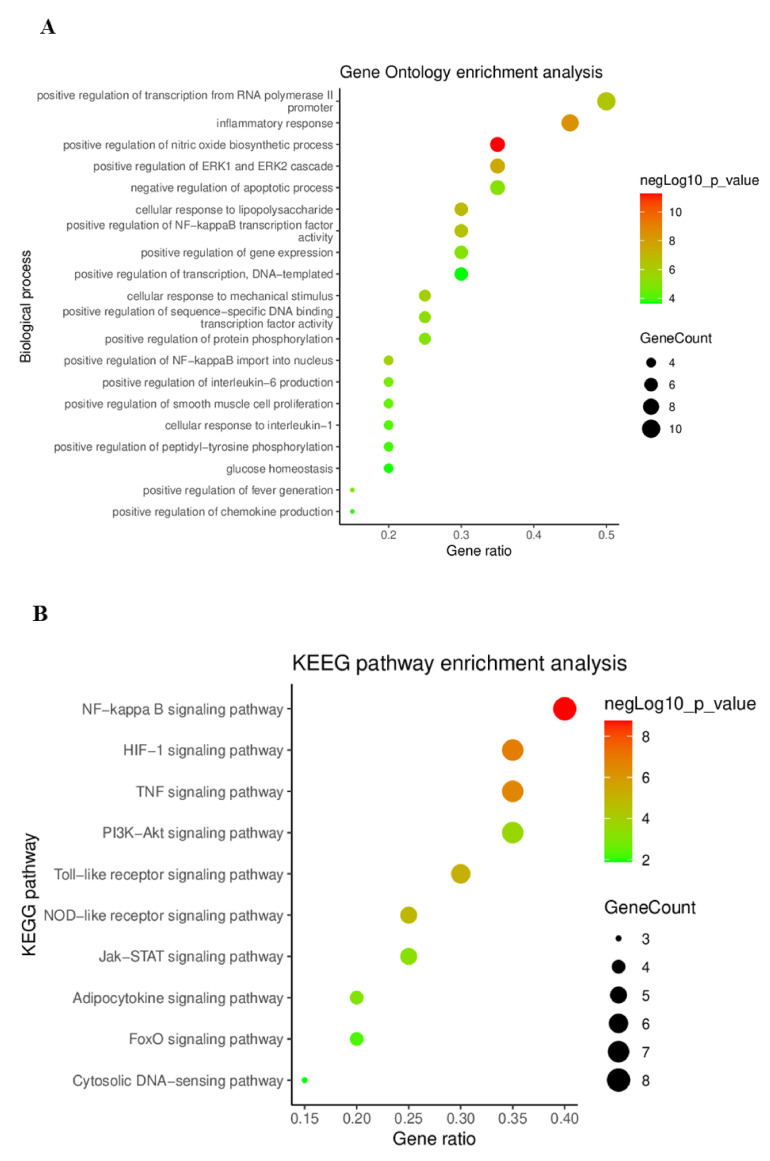
Bubble chart of GO and KEGG pathway enrichment analysis: (**A**) bubble chart of the GO enrichment analysis; (**B**) bubble chart of the KEGG pathway enrichment analysis.

GO and KEGG pathway enrichment analyses of key targets were performed using the DAVID database to identify related biological processes and signaling pathways. In the GO analysis, 130 GO terms were acquired, and the top 20 terms were selected by *p*-value. As shown in Figure 5A, the key targets were closely associated with inflammatory responses, including biosynthesis of the nitric oxide biosynthetic process and ERK1 and ERK2 cascades. In the KEGG pathway analysis, 52 KEGG pathway terms were acquired, and 10 top-ranking pathways were selected on the basis of their *p*-values. The selected 10 pathways are presented as a bubble chart in Figure 5B. KEGG pathway analysis showed that the key targets were associated with the NF-κB, HIF-1, and TNF signaling pathways. Specifically, the NF-κB signaling pathway exhibited the highest *p*-value and gene ratio. According to several studies, suppression of the NF-κB signaling pathway exhibits anti-gastritis effects [29,30,31]. In addition, extracts and derived compounds of *C. cassia* showed anti-inflammatory activities by suppressing the NF-κB signaling pathway [32,33,34]. Therefore, these results suggest that the key targets are highly associated with the inflammatory response and NF-κB signaling pathway.

#### 2.4.5. Analysis of Expected Active Compounds–Key Targets–Pathway (C-T-P) Network

**Figure 6:**
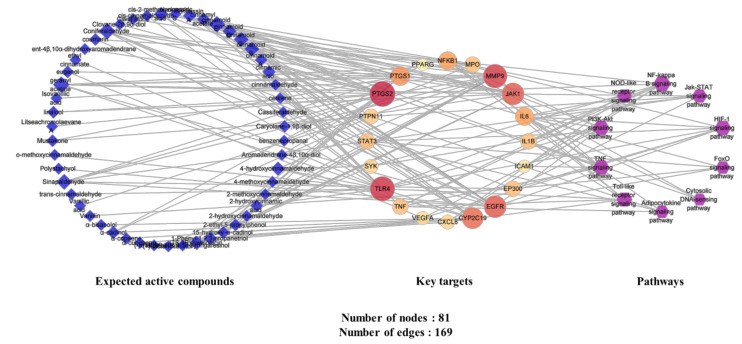
Expected active compounds–key targets–pathways (C-T-P) network.

A C-T-P network was constructed to provide a comprehensive interpretation of network pharmacology analysis. The C-T-P network consisted of 81 nodes (51 expected active compounds, 20 key targets, and 10 pathways) and 169 edges (Figure 6). The blue diamond nodes represent the expected active compounds, the reddish circle nodes indicate the key targets, and the purple hexagon nodes symbolize the pathways. The sizes of the nodes and colors of the key target nodes denote the degree. In other words, the larger and redder the key targets are, the higher the degree value they have, thereby indicating that they are important nodes in the network.

Among the 51 expected active compound nodes, 10 compounds were selected as key components of *C. cassia* on the basis of their degree values (Table 5). According to previous studies, 4 of the 10 key components have exhibited anti-inflammatory activities [35,36,37,38]. For example, geranyl acetate was found to suppress iNOS and COX-2 gene expression as well as LPS-induced IL-1β biosynthesis in macrophages [35]. Coniferaldehyde downregulates the expressions of iNOS, COX-2, and cell death [36]. Sinapaldehyde has inhibited the production of NO, ROS, TNF-α, and IL6 in LPS-stimulated cells [37]. Additionally, 4-hydroxycinnamaldehyde has inhibited NO production in LPS-induced cells [38]. These results suggest that key components are involved in the anti-inflammatory activities of *C. cassia*.

In the key target nodes, PTGS2, TLR4, MMP9, JAK1, EGFR, CYP2C19, and PTGS1 showed ≥10 degrees. Most of these are strongly associated with inflammatory response mechanisms. For example, PTGS2 is induced by inflammatory stimuli and is a target for nonsteroidal anti-inflammatory drugs (NSAIDs) [39]. TLR4 is a sensor receptor for LPS and is involved in inflammatory responses [40]. Additionally, MMP9 is induced by neutrophils and macrophages and is associated with various inflammatory diseases [41]. JAK1 is also strongly associated with the signal transduction of inflammatory cytokine receptors [42], and EGFR downregulates transcription factors such as NF-κB and stimulates pro-inflammatory gene transcription [43]. CYP2C19 is involved in the metabolism of several drugs. Specifically, proton pump inhibitors (PPIs), which are widely used for treating *H. pylori* infections and gastric ulcers, are mainly metabolized in the liver by CYP2C19, thereby affecting drug efficacy [44]. Therefore, these genes may be crucial targets for the anti-gastritis activities of the key components of *C. cassia*.

The NF-κB signaling pathway exhibited the highest degree of expression in the pathway nodes. This is one of the major inflammation-related pathways [45] and was predicted to be a critical pathway in the GO and KEGG pathway enrichment analyses. Furthermore, recent studies have revealed a correlation between the NF-κB signaling pathway and anti-gastritis activities [32,34]. Consequently, the network pharmacology analysis conducted in this study indicated that the anti-gastritis activity of *C. cassia* may be caused by its inhibitory effect on the NF-κB signaling pathway through the determined key components.

## 3. Discussion

Gastritis and gastric ulcers can develop in children to adults and have symptoms such as burning upper gastric pain, nausea, indigestion, and vomiting [46]. The causes of these symptoms are damage to the gastric mucosa by irregular eating habits, excessive drinking, stress, *Helicobacter pylori* infections, and nonsteroidal anti-inflammatory drugs (NSAIDs) [9]. NSAIDs have demonstrated antipyretic, analgesic, and anti-inflammatory effects that can reduce prostaglandins through inhibiting cyclooxygenase-1 (COX-1) and cyclooxygenase-2 (COX-2) [47]. However, contrary to this effect was another study observed that acute gastritis caused by damage to the gastric mucosa occurred in patients who took NSAIDs [48,49]. Indomethacin, an NSAID, is a drug used in an NSAID-induced acute gastritis model [50,51,52]. In this study, we observed the beneficial effects of *C. cassia* extracts on indomethacin-induced gastric damage in mice following oral administration of WCC, BCC, and ECC. The injured area of the gastric mucosa treated with ranitidine, AE (*Artemisia* extract), and *C. cassia* extracts showed a statistically significant decrease in the injured area compared to the indomethacin-induced control group. To confirm the dose-dependent effect of ECC on indomethacin-induced gastric damage, experimental animals were administered AE, which was used as a positive control, and ECC at concentrations of 50, 100, and 150 mg/kg to indomethacin-induced gastric injury sites. The gastric mucosa injury areas in the ranitidine-, AE-, and ECC-treated groups showed a statistically significant decrease compared to that in the indomethacin-induced control group. The protective effect of ECC was as strong as that of AE.

In addition, the effects of *C. cassia* extracts were tested in another model of gastric mucosal damage caused by an EtOH/HCl mixture, and EtOH is widely used in the acute gastritis model [53,54,55]. The mucosal damage mechanism in this model exacerbates gastritis by directly stimulating the gastric mucosa to increase the activity of free radicals and lipid peroxidation [56,57]. Two types of *C. cassia* extracts, WCC and ECC, were orally administered to treat gastric injuries caused by HCl-EtOH. The groups treated with ranitidine, WCC, and ECC decreased gastric mucosal damage compared to the control group, but the difference was not statistically significant. Additionally, in an experiment confirming the protective effects of the WCC and ECC administration groups against the 100% EtOH-induced gastric mucosal injury, the WCC- and ECC-treated groups inhibited the gastric mucosal damage compared to the control group, but there was no statistically significant difference between the two. Gastric mucosal damage induced by NSAIDs, EtOH, and EtOH/HCl is caused by direct stimulation of the mucosal surface as well as via the generation of ROS or induction of lipid peroxidation. Previous studies have shown that *C. cassia* has antioxidant and anti-inflammatory effects [58,59,60]. Although the protective effects of WCC and ECC on EtOH-induced gastritis were not statistically significant, they may provide protective effects against gastritis-inducing factors other than NSAIDs.

Damaged mucous membranes can cause indigestion, nausea, and heartburn, among other symptoms. The causes of damage are stress, irregular eating habits, overeating, and *Helicobacter pylori* infections [5,61]. In a stimulated stomach, secretion of gastric juice is promoted and pepsin is activated [62]. Pepsin is activated at a low pH (pH 1.8–2.0) to break down proteins, and continuous exposure of the gastric wall to gastric juice and pepsin causes further damage [63,64]. Gastric acid is one of the factors that causes gastritis [65], and regulating gastric pH can prevent damage to the gastric mucosa [66,67]. In evaluating the gastric acid inhibitory effect via oral administration of WCC and ECC for treating pyloric ligation and HCl-EtOH-induced gastric injury, the amount of gastric juice secretion in the control group was higher than that in the vehicle group. In the group treated with the *C. cassia* extract, the gastric juice secretion amount showed a significant difference compared to the control group, with 20% in the group treated with WCC and 14.3% in the group treated with ECC. The pH of the lansoprazole-administered group was 6.2, which was significantly higher than that of the control group; this was likely caused by the inhibition of gastric acid secretion. The pH of the WCC-administered group was 2.8, which was lower than that of the control group, and the pH of the ECC-administered group was 3.6, which was similar to that of the control group (Table 2). Thus, the effects of the *C. cassia* extract on pH ranged from weak to absent. Total gastric acidity in the lansoprazole-administered group was significantly lower than that in the control group. The total gastric acidity of the WCC-and ECC-administered groups decreased, but these values were not statistically significant compared to that of the control group. In a rat model of gastric injury induced by pyloric ligation and HCl-EtOH, administration of WCC and ECC had no effect on pH compared to the control group, but ECC exerted an inhibitory effect on gastric juice secretion.

In the present study, we used a network pharmacology analysis to predict the active compounds, key targets, and pharmacological mechanisms of *C. cassia* against gastritis. The results showed that 10 key components were predicted as anti-gastritis effect-related compounds of *C. cassia*, among 51 expected active compounds. Specifically, four of the selected 10 key components have shown anti-inflammatory activities in previous studies [35,36,37,38]. Additionally, inflammatory response-related genes, PTGS2, TLR4, MMP9, JAK1, and EGFR, showed a significant degree value in the C-T-P network. These target genes were highly associated with the inflammatory response in previous studies [39,40,41,42,43]. The NF-κB signaling pathway, a widely known inflammatory response mechanism, comprised a major signaling pathway within the network pharmacology analysis. Several studies have recently been conducted on the relationship between the NF-κB signaling pathway and gastritis [32,33,34]. These results suggest that the anti-gastritis activity of *C. cassia* may be induced via the anti-inflammatory effects of its key components through suppressing inflammation-related genes and signaling the pathways identified in this study.

In summary, this study confirmed that *C. cassia* extracts reduced the area of gastric mucosa injuries caused by indomethacin, NSAID, EtOH, and EtOH/HCl, and also decreased the amount of gastric acid and total gastric acidity in comparison with the damaged gastric mucosal control group. In addition, we inferred the active ingredients and predicted targets and mechanisms of action of the anti-gastritis activities of *C. cassia* through applying a network pharmacology analysis. According to the analysis of the C-T-P network, 10 compounds were selected as key components on the basis of their degree values. Among them, geranyl acetate, coniferaldehyde, sinapaldehyde, and 4-hydroxycinnamaldehye have demonstrated anti-inflammatory activities in previous studies. In addition, PTGS2, TRL4, MMP9, JAK1, EGFR, CYP2C19, and PTGS1 were identified as crucial anti-gastritis target genes in *C. cassia*. Importantly, the NF-κB signaling pathway was identified as the major signaling pathway responsible for the anti-gastritis effect of *C. cassia*. These results suggest that *C. cassia* can potentially help mitigate stomach damage. Furthermore, we considered that the mitigation effect on gastric injury was cocktail effect caused by the individual compounds in *C. cassia*.

## 4. Materials and Methods

### 4.1. Plant Material and Chemicals

The *C. cassia* extracts (WCC, BCC, ECC) and *Artemisia* extract (AE) used in the experiment were provided by Chong Kun Dang Research Institute, Korea [14]. Briefly, *C. cassia* was extracted with distilled water using a heat-reflux extractor for 5 h after non-processing or processing. The extract was concentrated using reduced pressure in the evaporator and dry-powdered.

### 4.2. Experimental Animals and Their Management

All experimental animals used male Sprague-Dawley rats (7 weeks old upon receipt, company, nation) were used for the experiments after a week of acclimation. During the acclimation and experimental period, the animals were divided into three rats per cage and kept at a constant temperature (20–25 °C) and humidity (30–35%) in a 12 h light/dark cycle. The rats were given ad libitum access to feed and water. In experiments for inducing gastric ulcers, mice were administered their respective *C. cassia* extract article (except for the gastritis control groups, which were administered a carboxymethyl cellulose sodium salt (CMC) solution) after 48 h of feed deprivation, and gastric damage was induced by administering indomethacin (80 mg/kg) [51,52], ethanol (100% EtOH), or an ethanol-HCl (150 mM HCl in 70% EtOH) [11,12] 30 min after the pre-treatment. Five hours later, all animals were sacrificed, and their stomachs were extracted for experimental purposes. The study was conducted according to the guidelines of the Declaration of Helsinki and approved by the Institutional Review Board of Inha University (protocol codes INHA120907-157, 158 approved on 9 September 2012, INHA130107-185 approved on 7 January 2013).

#### 4.2.1. Animal Experiment Design

Experimental animals were divided into three groups (10 individuals per group): (1) a gastric ulcer control group with gastric damage induced following administration of the CMC solution, (2) experimental groups with gastric damage induced following administration of the *C. cassia* extract (150 mg/kg) at different concentrations, and (3) positive control groups with gastric damage induced following administration of ranitidine (75 mg/kg). All samples were dissolved in a CMC solution and orally administered at a ratio of 10 mL per 1 kg animal weight.

#### 4.2.2. Determination of Stomach Injury

For evaluation of gastric mucosal damage, each stomach was removed after sacrificing the animals. The gastric mucosa pictures were taken at the same location using a digital camera (IXY digital 10, Canon, Tokyo, Japan) fixed to a stand. The stomachs of all the animals in each group were stretched to determine the degree of injury. The damaged area (ulcer index) was analyzed using ImageJ software (version 1.41, Bethesda, MD, USA) after taking a picture with a digital camera. The degree of gastric mucosa damage was calculated using the formula below, and the difference of gastric mucosal damage in each group was evaluated through statistical analysis.

Ulcer index (%) = (damaged area/total area of the stomach) × 100(1)

#### 4.2.3. Evaluation of Gastric Acid Secretion Inhibition

The gastric acid secretion inhibitory efficacy of the *C. cassia* extract articles was evaluated by measuring the gastric juice secretion rate, gastric juice pH, and total gastric acidity. This study was approved by the Institutional Animal Care and Use Committee (INHA130307-196, approved on 7 March 2013). Mice fasted for 48 h, and then *C. cassia* extract articles were administered 30 min before the HCl/EtOH mixture (150 mM HCl in 70% EtOH) induced acute gastric damage [11,12]. After 1 h, the pylorus was ligated, and the stomachs were removed after 4 h. The stomachs were removed, and gastric juices were collected via syringe. The supernatant was collected via centrifugation at 3000 rpm, and the volumes were measured. The pH of the gastric juice was measured using pH test paper. Acidity was measured as the amount in which the pH was neutralized by adding 0.01 N NaOH to the collected gastric juice, and total acidity was expressed as mEq/L. The total gastric acidity of the gastric juice secretion volume after 4 h was expressed as the calculated value.

Total gastric acidity [mEq/4 h] = (total acidity × gastric juice volume)/1000(2)

### 4.3. Network Pharmacology Analysis

#### 4.3.1. Collecting and Screening of Chemical Components of *C. cassia*

The chemical components of *C. cassia* were manually determined through a review of databases and the literature, including National Herbal Medicine Information (NHMI, https://nifds.go.kr/nhmi/main.do (accessed on 29 December 2021)) and PubMed (https://pubmed.ncbi.nlm.nih.gov/ (accessed on 29 December 2021)). All compounds were evaluated for their drug-likeness (DL) and oral bioavailability (OB) using the quantitative estimate of drug-likeness (QED) method. The physicochemical properties of the components were acquired from the SwissADME database (https://www.swissadme.ch/ (accessed on 29 December 2021)) [68] and were introduced into the QED function to calculate the QED value of each component [69]. Two cut-off values (QED ≥ 0.4 and OB = TRUE) were set to select the expected active compounds of *C. cassia*.

#### 4.3.2. Acquisition of Expected Active Compounds Targets and Disease Related Targets

SMILES codes of the expected active compounds of *C. cassia* were obtained from the PubChem (https://pubchem.ncbi.nlm.nih.gov/ (accessed on 29 December 2021)) and SciFinder-n (https://scifinder-n.cas.org/ (accessed on 29 December 2021)) databases and uploaded to the SwissTargetPrediction database (http://www.swisstargetprediction.ch/ (accessed on 29 December 2021)) [70] to obtain the predicted targets. Disease-related targets were detected using the GeneCards database (https://www.genecards.org/ (accessed on 29 December 2021)) [71]. “Gastritis” was used as a key word for searching the disease related targets.

#### 4.3.3. Acquisition of Potential Targets

Potential targets were selected as follows: (1) duplicates and false-positive targets of the expected active compounds were removed, then (2) compared with gastritis-related targets and the obtained common targets, and (3) a relevance score (≥1000) was set as the cut-off value to screen potential targets. The potential targets were visualized with a Venn diagram using Venny 2.1 (https://bioinfogp.cnb.csic.es/tools/venny/index.html (accessed on 29 December 2021)) [72]. The DisGeNET database (https://www.disgenet.org/search (accessed on 29 December 2021)) [73] was used to retrieve specific protein class information for potential targets.

#### 4.3.4. Construction and Analysis of the Protein-Protein Interaction (PPI) Network

Protein–protein interactions of potential targets were analyzed using the STRING database (https://string-db.org/ (accessed on 29 December 2021)) [74]. The analysis setting was set as follows: the required score was a high confidence (0.700) and a medium false discovery rate (FDR) stringency (5 percent). The resultant data were imported into Cytoscape software (v.3.9.0) (National Resource for Network Biology (NRNB), Bethesda, MD, USA) [75] to construct and analyze the PPI network of the potential targets. The “degree”, “betweenness centrality”, and “closeness centrality” parameters were used to estimate the topological features of the nodes in the network. Key targets were selected from the potential targets based on the topological analysis results.

#### 4.3.5. Gene Ontology (GO) and Kyoto Encyclopedia of Genes and Genomes (KEGG) Pathway Enrichment Analysis

GO and KEGG pathway enrichment analysis of the key targets were performed using the DAVID Bioinformatics Resources 6.8 database (https://david.ncifcrf.gov/home.jsp (accessed on 29 December 2021)) [76]. The false discovery rate (FDR) error control method (FDR < 0.05) was used to correct the *p*-value, and *p* < 0.05 was set as a threshold value to obtain biological processes and signaling pathways. The GO and KEGG pathway enrichment analysis results were visualized using ImageGP (EHBIO Gene Technology, Beijing, China) (http://www.ehbio.com/ImageGP (accessed on 29 December 2021)).

#### 4.3.6. Construction and Analysis of Expected Active Compounds-Key Targets-Pathways (C-T-P) Network

An integrated network of expected active compounds, key targets, and pathways was constructed and analyzed using the Cytoscape software (v.3.9.0) (NRNB, Bethesda, MD, USA).

### 4.4. Statistical Analysis

All numerical values are expressed as mean ± standard deviation. Statistical analysis was performed using one-way analysis of variance followed by Tukey’s post hoc test. This statistical analysis was performed using the SPSS software package for Windows (v.19, SPSS Inc., Chicago, IL, USA), and significant differences were considered present at the 5% level (*p* < 0.05).

## 5. Conclusions

The results of this study confirmed that *C. cassia* extracts reduced the area of gastric mucosa injuries caused by indomethacin, NSAID, EtOH, and EtOH/HCl. In addition, WCC and ECC decreased the amount of gastric acid and total gastric acidity in comparison with the control group. In this study, we inferred the active ingredients, predicted targets, and mechanisms of action of the anti-gastritis activities of *C. cassia* through applying a network pharmacology analysis. According to the analysis of the C-T-P network, 10 compounds were selected as key components on the basis of their degree values. In addition, PTGS2, TRL4, MMP9, JAK1, EGFR, CYP2C19, and PTGS1 were identified as crucial anti-gastritis target genes in *C. cassia*. Importantly, the NF-κB signaling pathway was identified as the major signaling pathway responsible for the anti-gastritis effect of *C. cassia*. These results suggest that *C. cassia* can potentially help mitigate stomach damage, and the mitigation effect on gastric injury was synergistic effects caused by the individual compounds in *C. cassia.*

## Figures and Tables

**Table 1:** Potential effects of *C. cassia* extracts against HCl-EtOH/EtOH induced damage.

Drug	Treatment	Dose (p.o.)	Ulcer Index (%)	Inhibition (%)
HCl-EtOH	Vehicle	-	8.44 ± 3.84	-
	Ranitidine	75 mg/kg	7.11 ± 4.23	15.78
	WCC	150 mg/kg	6.91 ± 4.59	18.09
	ECC	150 mg/kg	4.69 ± 3.39	44.46
EtOH	Vehicle	-	12.75 ± 4.65	-
	Ranitidine	75 mg/kg	20.15 ± 11.26	−58.32
	WCC	150 mg/kg	9.21 ± 8.58	27.74
	ECC	150 mg/kg	8.95 ± 5.74	29.74

Ulcer index data are expressed as mean ± SD (*n* = 10), and inhibition data were compared with the vehicle control percentage. Statistical analysis was performed using the SPSS Statistics 19.

**Table 2:** Potential effect of the *C. cassia* extract article against the volume, pH, and total acidity of gastric juice.

Treatment	Gastric Juice Volume (mL)	pH	Total Acidity (mEq/4 h)
Vehicle	0.6 ± 0.4	2.1 ± 0.8	0.040 ± 0.041
Control	3.5 ± 0.7	3.7 ± 1.0	0.112 ± 0.055
Lansoprazole	3.4 ± 0.7	6.2 ± 1.0 **	0.030 ± 0.018 **
WCC	2.8 ± 0.7	2.9 ± 0.9	0.101 ± 0.024
ECC	2.7 ± 0.6 *	3.6 ± 0.5	0.099 ± 0.028

Each treatment concentration was administered (lansoprazole: 30 mg/kg, WCC: 300 mg/kg). All data are expressed as mean ± SD (*n* = 10), and statistical analysis was performed using SPSS Statistics 19. * *p* < 0.05 compared to the control group. ** *p* < 0.01 compared to the control group. WCC: water extract of *C. cassia*; ECC: water extract of *C. cassia* prewashed ethyl acetate.

**Table 3:** List of potential targets.

No.	Uniprot ID	Gene	Relevance Score	Targets	Protein Class
1	Q09472	*EP300*	23.750	E1A-binding protein p300	-
2	P01584	*IL1B*	18.992	interleukin 1 beta	-
3	P01375	*TNF*	12.518	tumor necrosis factor	Signaling
4	P10145	*CXCL8*	11.503	C-X-C motif chemokine ligand 8	Signaling
5	P05231	*IL6*	10.297	interleukin 6	-
6	P35354	*PTGS2*	9.818	prostaglandin-endoperoxide synthase 2	Enzyme
7	O00206	*TLR4*	8.350	toll-like receptor 4	-
8	P35228	*NOS2*	7.650	nitric oxide synthase 2	-
9	P33261	*CYP2C19*	7.234	cytochrome P450 family 2 subfamily C member 19	-
10	P14174	*MIF*	6.687	macrophage migration inhibitory factor	-
11	P43405	*SYK*	6.451	spleen-associated tyrosine kinase	Kinase
12	P19838	*NFKB1*	6.449	nuclear factor kappa B subunit 1	Transcription factor
13	P54707	*ATP12A*	6.033	ATPase H+/K+ transporting non-gastric alpha2 subunit	Transporter
14	P23219	*PTGS1*	5.667	prostaglandin-endoperoxide synthase 1	Enzyme
15	P40763	*STAT3*	5.485	signal transducer and activator of transcription 3	Nucleic acid binding
16	O14684	*PTGES*	4.898	prostaglandin E synthase	-
17	P05091	*ALDH2*	4.614	aldehyde dehydrogenase 2 family member	Enzyme
18	P08581	*MET*	3.626	MET proto-oncogene, receptor tyrosine kinase	Kinase
19	Q05655	*PRKCD*	3.378	protein kinase C delta	Kinase
20	P05164	*MPO*	3.197	myeloperoxidase	Enzyme
21	P23458	*JAK1*	3.146	Janus kinase 1	Kinase
22	P00533	*EGFR*	2.978	epidermal growth factor receptor	Kinase
23	P08183	*ABCB1*	2.735	ATP binding cassette subfamily B member 1	Transporter
24	P14780	*MMP9*	2.670	matrix metallopeptidase 9	Enzyme
25	P10415	*BCL2*	2.352	BCL2 apoptosis regulator	Signaling
26	P08253	*MMP2*	2.344	matrix metallopeptidase 2	Enzyme
27	P39877	*PLA2G5*	2.251	phospholipase A2 group V	Enzyme
28	Q06124	*PTPN11*	1.951	protein tyrosine phosphatase non-receptor type 11	-
29	Q9UBK2	*PPARG*	1.897	PPARG coactivator 1 alpha	Transcription factor
30	P15692	*VEGFA*	1.827	vascular endothelial growth factor A	Signaling
31	P21980	*TGM2*	1.812	transglutaminase 2	Enzyme
32	P12821	*ACE*	1.704	angiotensin I-converting enzyme	Enzyme
33	Q8NER1	*TRPV1*	1.702	transient receptor potential cation channel subfamily V member 1	Ion channel
34	P09237	*MMP7*	1.669	matrix metallopeptidase 7	Enzyme
35	Q16236	*NFE2L2*	1.543	nuclear factor, erythroid 2-like 2	Enzyme
36	P05362	*ICAM1*	1.526	intercellular adhesion molecule 1	-
37	P09874	*PARP1*	1.324	poly(ADP-ribose) polymerase 1	-
38	P47989	*XDH*	1.295	xanthine dehydrogenase	Enzyme
39	P51681	*CCR5*	1.266	C-C motif chemokine receptor 5 (gene/pseudogene)	G-protein-coupled receptor
40	P49682	*CXCR3*	1.266	C-X-C motif chemokine receptor 3	G-protein-coupled receptor
41	P00813	*ADA*	1.263	adenosine deaminase	Enzyme
42	P16581	*SELE*	1.240	selectin E	-
43	P20701	*ITGAL*	1.181	integrin subunit alpha L	-
44	P25116	*F2R*	1.181	coagulation factor II thrombin receptor	G-protein-coupled receptor
45	P26010	*ITGB7*	1.179	integrin subunit beta 7	Receptor
46	P08684	*CYP3A4*	1.164	cytochrome P450 family 3 subfamily A member 4	Enzyme
47	P10721	*KIT*	1.157	KIT proto-oncogene, receptor tyrosine kinase	Kinase
48	P22894	*MMP8*	1.131	matrix metallopeptidase 8	Enzyme
49	P51679	*CCR4*	1.131	C-C motif chemokine receptor 4	G-protein-coupled receptor
50	P15056	*BRAF*	1.103	B-Raf proto-oncogene, serine/threonine kinase	Kinase
51	P08246	*ELANE*	1.103	elastase, neutrophil-expressed	Enzyme
52	P29597	*TYK2*	1.081	tyrosine kinase 2	Kinase
53	Q08881	*ITK*	1.074	IL2-inducible T cell kinase	Kinase
54	P41180	*CASR*	1.074	calcium-sensing receptor	G-protein-coupled receptor
55	P05093	*CYP17A1*	1.074	cytochrome P450 family 17 subfamily A member 1	-
56	P05177	*CYP1A2*	1.074	cytochrome P450 family 1 subfamily A member 2	Enzyme
57	P14222	*PRF1*	1.074	perforin 1	-
58	P29274	*ADORA2A*	1.070	adenosine A2a receptor	G-protein-coupled receptor
59	P42345	*MTOR*	1.042	mechanistic target of rapamycin kinase	Kinase

**Table 4:** Key targets based on PPI network topological analysis.

No.	Uniprot ID	Gene	Relevance Score	Degree	Betweenness Centrality	Closeness Centrality
1	P40763	*STAT3*	5.485	16	0.079	0.864
2	P05231	*IL6*	10.297	15	0.057	0.826
3	P01375	*TNF*	12.503	15	0.058	0.826
4	P01584	*IL1B*	18.992	13	0.026	0.760
5	O00206	*TLR4*	8.350	13	0.041	0.760
6	P35354	*PTGS2*	4.898	13	0.166	0.760
7	P10145	*CXCL8*	11.503	12	0.019	0.731
8	P05362	*ICAM1*	1.526	12	0.031	0.731
9	P14780	*MMP9*	2.670	11	0.007	0.704
10	P15692	*VEGFA*	1.827	11	0.020	0.704
11	P19838	*NFKB1*	6.449	9	0.015	0.613
12	P00533	*EGFR*	2.978	9	0.012	0.655
13	P05164	*MPO*	3.197	8	0.031	0.633
14	Q06124	*PTPN11*	1.951	8	0.009	0.594
15	Q09472	*EP300*	23.750	7	0.003	0.576
16	P37231	*PPARG*	1.897	7	0.006	0.613
17	P23458	*JAK1*	3.146	6	0.004	0.559
18	P43405	*SYK*	6.451	6	0.006	0.559
19	P23219	*PTGS1*	5.667	3	0.007	0.475
20	P33261	*CYP2C19*	7.234	2	0.000	0.463

**Table 5:** List of 10 key components from the C-T-P network analysis.

Compound Name	Structure	Formula	Degree
Geranyl acetate	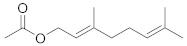	C_12_H_20_O_2_	7
Coniferaldehyde	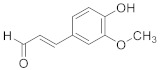	C_10_H_10_O_3_	7
Cinnamoid E	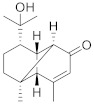	C_15_H_22_O_2_	7
Sinapaldehyde	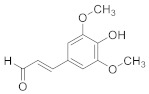	C_11_H_12_O_4_	6
Cinnamoid C	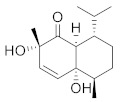	C_15_H_24_O_3_	5
Cinnamoid B	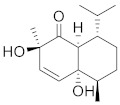	C_15_H_24_O_3_	5
4-Hydroxycinnamaldehyde	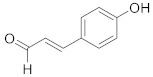	C_9_H_8_O_2_	4
(7S,8R)-Lawsonicin	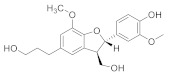	C_20_H_24_O_6_	4
cis-2-Methoxycinnamic acid	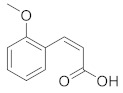	C_10_H_10_O_3_	4
Cinnamoid D	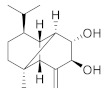	C_15_H_24_O_2_	4

## Data Availability

Data is contained within the article.

## Supplementary Materials

The following supporting information can be downloaded at https://www.mdpi.com/article/10.3390/plants11060716/s1. Table S1: List of physicochemical properties, QED, and OB of compounds from *C. cassia*. Table S2: List of expected active compounds from *C. cassia*.
