# Supplementary material for: Mitigation of Gastric Damage Using Cinnamomum cassia Extract: Network Pharmacological Analysis of Active Compounds and Protection Effects in Rats
Source: Plants (Basel). 2022 Mar 8;11(6):716. doi: 10.3390/plants11060716 (PMC8949351; doi:10.3390/plants11060716)
Supplement: Supplementary file 1 [file plants-11-00716-s001.zip › plants-1618644-supplementary.pdf]

plants

MDPI

Supplementary Materials

Mitigation of gastric damage using *Cinnamomum cassia* extract: Network pharmacological analysis of active compounds and protection effects in rats

Ji Hwan Lee [1,†], Hee Jae Kwak[2,†], Dongchul Shin [1], Hye Jin Seo [3], Shin Jung Park [3], Bo-Hee Hong [3], Myoung-Sook Shin [1], Seung Hyun Kim[2,*], Ki Sung Kang [1,*]

[1] College of Korean Medicine, Gachon University, Seongnam, 13120, Republic of Korea; kleert26@gmail.com (J.H.L), sdc2510@gmail.com (D.S.)
[2] College of Pharmacy, Yonsei Institute of Pharmaceutical Sciences, Yonsei University, Incheon, Republic of Korea; moon3685@naver.com (H.J.K.), kimsh11@yonsei.ac.kr (S.H.K.)
[3] Chong Kun Dang (CKD) Pharm Research Institute, Yongin-si, 16995, Republic of Korea
* Correspondence: Ki Sung Kang; Tel.: +82-31-750-5402; e-mail: kkang@gachon.ac.kr; 00000; Tel.: +82-32-749-4514; e-mail: kimsh11@yonsei.ac.kr.
† These authors contributed equally to this work.

Tables

Table S1. List of physicochemical properties, QED, and OB of compounds from *C. cassia*
Table S2. List of expected active compounds from *C. cassia*

Table S1. List of physicochemical properties, QED, and OB of compounds from *C. cassia*

No.	Compound Name	MW	ALOGP	HBA	HBD	PSA	ROTB	AROM	ALERTS	QED	OB
1	α-terpineol	154.25	2.58	1	1	20.23	1	0	1	0.575	TRUE
2	β-bisabolene	204.35	4.83	0	0	0.00	4	0	1	0.575	TRUE
3	α-bisabolol	222.37	3.76	1	1	20.23	4	0	1	0.718	TRUE
4	linalool	154.25	2.66	1	1	20.23	4	0	1	0.617	TRUE
5	camphene	136.23	3.43	0	0	0.00	0	0	1	0.447	TRUE
6	β-Pinene	136.23	3.42	0	0	0.00	0	0	1	0.447	TRUE
7	camphor	152.23	2.37	1	0	17.07	0	0	0	0.521	TRUE
8	geranyl acetate	196.29	3.21	2	0	26.30	6	0	1	0.610	TRUE
9	cinnzeylanol	384.46	0.54	7	6	95.45	1	0	0	0.393	TRUE
10	anhydrocinnzeylanol	366.45	1.36	6	4	107.22	1	0	1	0.522	TRUE
11	cinnzeylanone	382.45	0.37	7	5	127.45	1	0	0	0.443	TRUE
12	anhydrocinnzeylanine	408.49	1.44	7	3	113.29	3	0	2	0.478	TRUE
13	cinncassiol A 19-O-β-D-glucopyranoside	544.59	-1.04	12	8	206.60	5	0	1	0.195	FALSE
14	cinncassiol A	382.45	0.52	7	5	127.45	2	0	1	0.441	TRUE
15	cinncassiol B	400.46	-0.05	8	7	150.84	2	0	0	0.341	FALSE
16	cinncassiol C	380.43	0.46	7	4	124.29	2	0	0	0.549	TRUE
17	cinncassiol E	398.45	-0.22	8	6	139.84	1	0	0	0.356	FALSE
18	cinncassiol F	398.45	0.4	8	5	136.68	1	0	0	0.428	FALSE
19	cinncassiol G	382.45	0.48	7	5	127.45	2	0	1	0.440	TRUE
20	16-O-β-D-glucopyranosyl-19-deoxycinncassiol G	544.59	-0.87	12	8	206.60	5	0	1	0.199	FALSE
21	cinnacasol	382.45	0.52	7	5	127.45	2	0	1	0.441	TRUE
22	perseanol	384.46	0.19	7	6	130.61	1	0	0	0.380	FALSE
23	cinncassiol D1	352.47	1.69	5	4	90.15	2	0	0	0.607	TRUE
24	cinncassiol D1 glucoside	514.61	0.03	10	7	169.30	5	0	0	0.266	FALSE
25	cinncassiol D2	368.46	0.96	6	5	110.38	2	0	0	0.497	TRUE
26	cinncassiol D3	368.46	0.87	6	5	110.38	2	0	0	0.495	TRUE
27	18-hydroxyperseanol	400.46	-0.96	8	7	150.84	1	0	0	0.297	FALSE
28	caryophyllene	204.35	4.24	0	0	0.00	0	0	1	0.518	TRUE
29	α-cubebene	204.35	4.31	0	0	0.00	1	0	1	0.558	TRUE
30	(-)-isoledene	204.35	4.26	0	0	0.00	0	0	1	0.517	TRUE
31	patchouli alcohol	222.37	3.56	1	1	20.23	0	0	0	0.664	TRUE

32	α-copaene	204.35	4.3	0	0	0.00	1	0	1	0.559	TRUE
33	α-muurolene	204.35	4.08	0	0	0.00	1	0	1	0.565	TRUE
34	α-cadinol	222.37	3.43	1	1	20.23	1	0	1	0.674	TRUE
35	1-(1,5-dimethyl-4-hexenyl)-4-methylbenzene	202.34	4.86	0	0	0.00	4	1	1	0.612	TRUE
36	cedrene	204.35	4.36	0	0	0.00	0	0	1	0.514	TRUE
37	α-calacorene	200.32	4.38	0	0	0.00	1	1	0	0.631	TRUE
38	cinnamoid A	238.37	2.67	2	2	40.46	1	0	0	0.737	TRUE
39	cinnamoid B	252.35	2.08	3	2	57.53	1	1	1	0.752	TRUE
40	cinnamoid C	252.35	2.08	3	2	57.53	1	1	1	0.752	TRUE
41	cinnamoid D	236.35	2.37	2	2	40.46	1	0	1	0.686	TRUE
42	cinnamoid E	234.33	2.47	2	1	37.30	1	0	0	0.756	TRUE
43	15-hydroxy-α-cadinol	238.37	2.58	2	2	40.46	2	0	1	0.726	TRUE
44	ent-4β,10α-dihydroxyaromadendrane	238.37	2.59	2	2	40.46	0	0	0	0.681	TRUE
45	cinnamaldehyde	132.16	1.97	1	0	17.07	2	1	2	0.444	TRUE
46	cis-2-methoxycinnamic acid	178.18	1.71	3	1	46.53	3	1	1	0.716	TRUE
47	*o*-methoxycinnamaldehyde	162.19	2.03	2	0	26.30	3	1	2	0.502	TRUE
48	2-methoxycinnamaldehyde	162.19	2.03	2	0	26.30	3	1	2	0.502	TRUE
49	cinnamyl alcohol	134.18	1.96	1	1	20.23	2	1	0	0.655	TRUE
50	cis-cinnamaldehyde	132.16	1.97	1	0	17.07	2	1	2	0.444	TRUE
51	trans-cinnamaldehyde	132.16	1.97	1	0	17.07	2	1	2	0.444	TRUE
52	ethyl cinnamate	176.21	2.49	2	0	26.30	4	1	1	0.659	TRUE
53	eugenol	164.2	2.25	2	1	29.46	3	1	1	0.694	TRUE
54	cinnamyl acetate	176.21	2.33	2	0	26.30	4	1	0	0.703	TRUE
55	2-hydroxycinnamic acid	164.16	1.4	3	2	57.53	2	1	1	0.649	TRUE
56	2-hydroxycinnamaldehyde	148.16	1.63	2	1	37.30	2	1	2	0.511	TRUE
57	4-methoxycinnamaldehyde	162.19	1.95	2	0	26.30	3	1	2	0.501	TRUE
58	cinnamic acid	148.16	1.79	2	1	37.30	2	1	1	0.649	TRUE
59	cinnacasside A	512.55	0.58	11	6	175.37	9	1	0	0.273	FALSE
60	cinnacasside C	512.55	0.58	11	6	175.37	9	1	0	0.273	FALSE
61	cinnacasside B	512.55	0.58	11	6	175.37	9	1	0	0.273	FALSE
62	cinnacassoside D	330.33	-1.34	8	6	139.84	6	1	0	0.377	FALSE
63	cinnacassoside A	540.56	0.13	12	8	198.76	12	2	0	0.181	FALSE
64	cinnacassoside B	654.66	-0.32	15	8	226.45	13	2	0	0.139	FALSE

65	cinnacassoside C	464.42	-1.82	13	7	196.99	8	1	0	0.227	FALSE
66	cinncassin E	404.41	1.82	8	3	114.68	10	2	2	0.406	TRUE
67	cinncassin D	508.52	2.7	9	3	123.91	8	3	1	0.394	TRUE
68	picrasmalignan A	534.55	3.1	9	3	123.91	9	3	2	0.281	TRUE
69	(+)-leptolepisol C	498.52	2.42	9	5	138.07	9	3	0	0.301	FALSE
70	(-)-(7R,8S,7′R,8′S)-syringaresinol	418.44	2.33	8	2	95.84	6	2	0	0.738	TRUE
71	(+)-isolariciresinol	360.4	2.02	6	4	99.38	5	2	0	0.651	TRUE
72	(-)-secroisolariciresinol	362.42	2.5	6	4	99.38	9	2	0	0.547	TRUE
73	(7S,8R)-guaiacylglycerol-β-coniferyl aldehyde ether	374.38	1.95	7	3	105.45	9	2	2	0.455	TRUE
74	(7S,8R)-lawsonicin	360.4	2.36	6	3	88.38	7	2	0	0.703	TRUE
75	5′-methoxylariciresinol	390.43	2.34	7	3	97.61	7	2	0	0.668	TRUE
76	(+)-(7′R,8R,8′R)-5,5′-dimethoxylariciresinol	420.45	2.32	8	3	106.84	8	2	0	0.597	TRUE
77	2-ethyl-5-propylphenol	164.24	3.12	1	1	20.23	3	1	0	0.727	TRUE
78	3,4-dimethoxyphenethyl alcohol	182.22	1.54	3	1	38.69	4	1	0	0.767	TRUE
79	benzaldehyde	106.12	1.57	1	0	17.07	1	1	0	0.531	TRUE
80	phenylethyl alcohol	122.16	1.64	1	1	37.38	2	1	0	0.636	TRUE
81	benzenepropanal	134.18	1.91	1	0	17.07	3	1	1	0.577	TRUE
82	acetophenone	120.15	1.82	1	0	17.07	1	1	0	0.552	TRUE
83	styrene	104.15	2.72	0	0	0.00	1	1	0	0.514	TRUE
84	rosavin	428.43	-1.07	10	6	158.30	7	1	0	0.316	FALSE
85	coumarin	146.14	1.82	2	0	30.21	0	1	1	0.522	TRUE
86	evofolin B	318.32	1.87	6	3	96.22	6	2	0	0.752	TRUE
87	cinncassin C	434.44	2.19	9	3	123.91	8	2	0	0.573	TRUE
88	cinncassin B	392.4	1.8	8	3	106.84	6	2	0	0.683	TRUE
89	Epianhydrocinnzeylanol	366.45	1.36	6	4	107.22	1	0	1	0.522	TRUE
90	Cinnacasiol H	382.45	0.56	7	5	127.45	1	0	1	0.419	TRUE
91	Caryolane-1,9β-diol	238.37	2.75	2	2	40.46	0	0	0	0.681	TRUE
92	Clovane-2β,9α-diol	238.37	2.79	2	2	40.46	0	0	0	0.681	TRUE
93	Mustakone	218.33	3.36	1	0	17.07	1	0	0	0.659	TRUE
94	Aromadendrane-4β,10α-diol	238.37	2.59	2	2	40.46	0	0	0	0.681	TRUE
95	Litseachromolaevane A	234.33	3.37	2	1	37.30	5	1	0	0.848	TRUE
96	Coniferaldehyde	178.18	1.56	3	1	46.53	3	1	2	0.564	TRUE
97	Cassiferaldehyde	178.18	1.55	3	1	46.53	3	1	2	0.564	TRUE

98	Sinapaldehyde	208.21	1.54	4	1	55.76	4	1	2	0.602	TRUE
99	1-Phenyl-1,2,3-propanetriol	168.19	0.42	3	3	60.69	3	1	0	0.613	TRUE
100	cinncassin A	286.28	1.91	5	3	86.99	2	2	1	0.734	TRUE
101	(+)-Syringaresinol	418.44	2.33	8	2	95.84	6	2	0	0.738	TRUE
102	Pinoresinol	358.39	2.26	6	2	77.38	4	2	0	0.873	TRUE
103	(-)-Isolariciresinol	360.4	2.02	6	4	99.38	5	2	0	0.651	TRUE
104	Polystachyol	420.45	2.04	8	4	117.84	7	2	0	0.538	TRUE
105	Lariciresinol	360.4	2.38	6	3	88.38	6	2	0	0.733	TRUE
106	Ciwujiatone	434.44	1.78	9	3	123.91	8	2	0	0.570	TRUE
107	(-)-Secoisolariciresinol	362.42	2.5	6	4	99.38	9	2	0	0.547	TRUE
108	Syringaldehyde	182.17	0.93	4	1	55.76	3	1	0	0.755	TRUE
109	Vanillin	152.15	1.2	3	1	46.53	2	1	1	0.647	TRUE
110	Protocatechualdehyde	138.12	0.8	3	2	57.53	1	1	2	0.445	TRUE
111	Vanillic acid	168.15	1.08	4	2	66.76	2	1	0	0.693	TRUE
112	Isovanillic acid	168.15	0.98	4	2	66.76	2	1	0	0.690	TRUE
113	cis-4-Hydroxymellein	194.18	1.06	4	2	66.76	0	1	0	0.648	TRUE
114	4-hydroxycinnamaldehyde	148.16	1.58	2	2	37.30	2	1	2	0.62	TRUE

Table S2. List of expected active compounds from *C. cassia*

No.	Compound Name	MW	ALOGP	HBA	HBD	PSA	ROTB	AROM	ALERTS	QED	OB
1	α-terpineol	154.25	2.58	1	1	20.23	1	0	1	0.575	TRUE
2	β-bisabolene	204.35	4.83	0	0	0.00	4	0	1	0.575	TRUE
3	α-bisabolol	222.37	3.76	1	1	20.23	4	0	1	0.718	TRUE
4	linalool	154.25	2.66	1	1	20.23	4	0	1	0.617	TRUE
5	camphene	136.23	3.43	0	0	0.00	0	0	1	0.447	TRUE
6	β-Pinene	136.23	3.42	0	0	0.00	0	0	1	0.447	TRUE
7	camphor	152.23	2.37	1	0	17.07	0	0	0	0.521	TRUE
8	geranyl acetate	196.29	3.21	2	0	26.30	6	0	1	0.610	TRUE
9	anhydrocinnzeylanol	366.45	1.36	6	4	107.22	1	0	1	0.522	TRUE
10	cinnzeylanone	382.45	0.37	7	5	127.45	1	0	0	0.443	TRUE
11	anhydrocinnzeylanine	408.49	1.44	7	3	113.29	3	0	2	0.478	TRUE
12	cinncassiol A	382.45	0.52	7	5	127.45	2	0	1	0.441	TRUE
13	cinncassiol C	380.43	0.46	7	4	124.29	2	0	0	0.549	TRUE
14	cinncassiol G	382.45	0.48	7	5	127.45	2	0	1	0.440	TRUE
15	cinnacasol	382.45	0.52	7	5	127.45	2	0	1	0.441	TRUE
16	cinncassiol D1	352.47	1.69	5	4	90.15	2	0	0	0.607	TRUE
17	cinncassiol D2	368.46	0.96	6	5	110.38	2	0	0	0.497	TRUE
18	cinncassiol D3	368.46	0.87	6	5	110.38	2	0	0	0.495	TRUE
19	caryophyllene	204.35	4.24	0	0	0.00	0	0	1	0.518	TRUE
20	α-cubebene	204.35	4.31	0	0	0.00	1	0	1	0.558	TRUE
21	(-)-isoledene	204.35	4.26	0	0	0.00	0	0	1	0.517	TRUE
22	patchouli alcohol	222.37	3.56	1	1	20.23	0	0	0	0.664	TRUE
23	α-copaene	204.35	4.30	0	0	0.00	1	0	1	0.559	TRUE
24	α-muurolene	204.35	4.08	0	0	0.00	1	0	1	0.565	TRUE
25	α-cadinol	222.37	3.43	1	1	20.23	1	0	1	0.674	TRUE
26	1-(1,5-dimethyl-4-hexenyl)-4-methylbenzene	202.34	4.86	0	0	0.00	4	1	1	0.612	TRUE
27	cedrene	204.35	4.36	0	0	0.00	0	0	1	0.514	TRUE
28	α-calacorene	200.32	4.38	0	0	0.00	1	1	0	0.631	TRUE

29	cinnamoid A	238.37	2.67	2	2	40.46	1	0	0	0.737	TRUE
30	cinnamoid B	252.35	2.08	3	2	57.53	1	1	1	0.752	TRUE
31	cinnamoid C	252.35	2.08	3	2	57.53	1	1	1	0.752	TRUE
32	cinnamoid D	236.35	2.37	2	2	40.46	1	0	1	0.686	TRUE
33	cinnamoid E	234.33	2.47	2	1	37.30	1	0	0	0.756	TRUE
34	15-hydroxy-α-cadinol	238.37	2.58	2	2	40.46	2	0	1	0.726	TRUE
35	ent-4β,10α-dihydroxyaromadendrane	238.37	2.59	2	2	40.46	0	0	0	0.681	TRUE
36	cinnamaldehyde	132.16	1.97	1	0	17.07	2	1	2	0.444	TRUE
37	cis-2-methoxycinnamic acid	178.18	1.71	3	1	46.53	3	1	1	0.716	TRUE
38	*o*-methoxycinnamaldehyde	162.19	2.03	2	0	26.30	3	1	2	0.502	TRUE
39	2-methoxycinnamaldehyde	162.19	2.03	2	0	26.30	3	1	2	0.502	TRUE
40	cinnamyl alcohol	134.18	1.96	1	1	20.23	2	1	0	0.655	TRUE
41	cis-cinnamaldehyde	132.16	1.97	1	0	17.07	2	1	2	0.444	TRUE
42	trans-cinnamaldehyde	132.16	1.97	1	0	17.07	2	1	2	0.444	TRUE
43	ethyl cinnamate	176.21	2.49	2	0	26.30	4	1	1	0.659	TRUE
44	eugenol	164.2	2.25	2	1	29.46	3	1	1	0.694	TRUE
45	cinnamyl acetate	176.21	2.33	2	0	26.30	4	1	0	0.703	TRUE
46	2-hydroxycinnamic acid	164.16	1.40	3	2	57.53	2	1	1	0.649	TRUE
47	2-hydroxycinnamaldehyde	148.16	1.63	2	1	37.30	2	1	2	0.511	TRUE
48	4-methoxycinnamaldehyde	162.19	1.95	2	0	26.30	3	1	2	0.501	TRUE
49	cinnamic acid	148.16	1.79	2	1	37.30	2	1	1	0.649	TRUE
50	cinncassin E	404.41	1.82	8	3	114.68	10	2	2	0.406	TRUE
51	(-)-(7R,8S,7′R,8′S)-syringaresinol	418.44	2.33	8	2	95.84	6	2	0	0.738	TRUE
52	(+)-isolariciresinol	360.4	2.02	6	4	99.38	5	2	0	0.651	TRUE
53	(-)-secroisolariciresinol	362.42	2.50	6	4	99.38	9	2	0	0.547	TRUE
54	(7S,8R)-guaiacylglycerol-β-coniferyl aldehyde ether	374.38	1.95	7	3	105.45	9	2	2	0.455	TRUE
55	(7S,8R)-lawsonicin	360.4	2.36	6	3	88.38	7	2	0	0.703	TRUE
56	5′-methoxylariciresinol	390.43	2.34	7	3	97.61	7	2	0	0.668	TRUE
57	(+)-(7′R,8R,8′R)-5,5′-dimethoxylariciresinol	420.45	2.32	8	3	106.84	8	2	0	0.597	TRUE
58	2-ethyl-5-propylphenol	164.24	3.12	1	1	20.23	3	1	0	0.727	TRUE

59	3,4-dimethoxyphenethyl alcohol	182.22	1.54	3	1	38.69	4	1	0	0.767	TRUE
60	benzaldehyde	106.12	1.57	1	0	17.07	1	1	0	0.531	TRUE
61	phenylethyl alcohol	122.16	1.64	1	1	37.38	2	1	0	0.636	TRUE
62	benzenepropanal	134.18	1.91	1	0	17.07	3	1	1	0.577	TRUE
63	acetophenone	120.15	1.82	1	0	17.07	1	1	0	0.552	TRUE
64	styrene	104.15	2.72	0	0	0.00	1	1	0	0.514	TRUE
65	coumarin	146.14	1.82	2	0	30.21	0	1	1	0.522	TRUE
66	evofolin B	318.32	1.87	6	3	96.22	6	2	0	0.752	TRUE
67	cinncassin C	434.44	2.19	9	3	123.91	8	2	0	0.573	TRUE
68	cinncassin B	392.4	1.80	8	3	106.84	6	2	0	0.683	TRUE
69	Epianhydrocinnzeylanol	366.45	1.36	6	4	107.22	1	0	1	0.522	TRUE
70	Cinnacasiol H	382.45	0.56	7	5	127.45	1	0	1	0.419	TRUE
71	Caryolane-1,9β-diol	238.37	2.75	2	2	40.46	0	0	0	0.681	TRUE
72	Clovane-2β,9α-diol	238.37	2.79	2	2	40.46	0	0	0	0.681	TRUE
73	Mustakone	218.33	3.36	1	0	17.07	1	0	0	0.659	TRUE
74	Aromadendrane-4β,10α-diol	238.37	2.59	2	2	40.46	0	0	0	0.681	TRUE
75	Litseachromolaevane A	234.33	3.37	2	1	37.30	5	1	0	0.848	TRUE
76	Coniferaldehyde	178.18	1.56	3	1	46.53	3	1	2	0.564	TRUE
77	Cassiferaldehyde	178.18	1.55	3	1	46.53	3	1	2	0.564	TRUE
78	Sinapaldehyde	208.21	1.54	4	1	55.76	4	1	2	0.602	TRUE
79	1-Phenyl-1,2,3-propanetriol	168.19	0.42	3	3	60.69	3	1	0	0.613	TRUE
80	cinncassin A	286.28	1.91	5	3	86.99	2	2	1	0.734	TRUE
81	(+)-Syringaresinol	418.44	2.33	8	2	95.84	6	2	0	0.738	TRUE
82	Pinoresinol	358.39	2.26	6	2	77.38	4	2	0	0.873	TRUE
83	(-)-Isolariciresinol	360.4	2.02	6	4	99.38	5	2	0	0.651	TRUE
84	Polystachyol	420.45	2.04	8	4	117.84	7	2	0	0.538	TRUE
85	Lariciresinol	360.4	2.38	6	3	88.38	6	2	0	0.733	TRUE
86	Ciwujiatone	434.44	1.78	9	3	123.91	8	2	0	0.570	TRUE
87	(-)-Secoisolariciresinol	362.42	2.50	6	4	99.38	9	2	0	0.547	TRUE
88	Syringaldehyde	182.17	0.93	4	1	55.76	3	1	0	0.755	TRUE

89	Vanillin	152.15	1.20	3	1	46.53	2	1	1	0.647	TRUE
90	Protocatechualdehyde	138.12	0.80	3	2	57.53	1	1	2	0.445	TRUE
91	Vanillic acid	168.15	1.08	4	2	66.76	2	1	0	0.693	TRUE
92	Isovanillic acid	168.15	0.98	4	2	66.76	2	1	0	0.690	TRUE
93	cis-4-Hydroxymellein	194.18	1.06	4	2	66.76	0	1	0	0.648	TRUE
94	4-hydroxycinnamaldehyde	148.16	1.58	2	2	37.30	2	1	2	0.620	TRUE
